# Genetic selection? A study of individual variation in the enzymes of folate metabolism

**DOI:** 10.1186/1471-2350-11-18

**Published:** 2010-02-01

**Authors:** Barbara A Jennings, Gavin A Willis, Jane Skinner, Caroline L Relton

**Affiliations:** 1 School of Medicine, Health Policy and Practice, University of East Anglia, Norwich NR4 7TJ, UK; 2 Department of Molecular Genetics, Norfolk and Norwich University Hospital, Norwich NR4 7UY, UK; 3 Institute for Ageing and Health and Human Nutrition Research Centre, Newcastle University, Royal Victoria Infirmary, Newcastle upon Tyne, NE1 4LP, UK

## Abstract

**Background:**

Genetic variation in folate metabolism has been associated with survival *in utero*, the success of *in vitro *fertilisation, multiple pathologies and longevity.

**Methods:**

We have looked at the prevalence of genetic variants of the enzymes MTHFR and TYMS in 2,898 DNA samples derived from five cohorts collected in the United Kingdom. The simultaneous analysis of genetic variants of the *MTHFR *and *TYMS *loci was carried out to investigate a putative gene-gene interaction that was first observed in an elderly male population from Norfolk.

**Results:**

We have made a consistent observation in five population cohorts; the proportion of individuals who are homozygous for the 2R allele of the 5'UTR *TYMS *polymorphism is less in individuals who are homozygous for the T allele of *MTHFR *677 than in individuals homozygous for the C allele of *MTHFR *677 (p = 0.02).

**Conclusions:**

These data may suggest a gene-gene interaction and could be evidence of genetic selection, with some pregnancies more or less viable as a consequence of genetic variation. If these genetic phenomena affect the way folate is handled at the cellular level *in utero *it is possible that maternal folic acid intake may over-ride such genetic selection.

## Background

Folate-dependent one-carbon metabolism is essential for nucleotide synthesis, methionine synthesis and for DNA methylation and depends on a number of enzymes that are functionally polymorphic. SNPs and other polymorphisms of the genes *MTHFR*, *MTRR *and *TYMS *have been associated with cardiovascular disease, neural tube defects, Down syndrome and leukaemia [1-4]. Adequate dietary folate, and its efficient metabolism have well-established roles in disease prevention; deficiency is associated with increased risk of neural tube defects, vascular disease, cancer and anaemia [5].

Proposals for the mandatory fortification of flour with folate in the United Kingdom are driven by the importance of this B vitamin to the development of the early embryo [6]. Embryo development and viability has been the subject of many epidemiological studies of genetic variants, including, and possibly predominantly, in those genes involved in folate metabolism [2-4,7]. B-vitamin status and folate metabolism have been shown to influence the outcome of *in vitro *fertilisation (IVF) [8,9]. Women who are homozygous for a variant of the gene *MTHFR *(1298A>C) were less likely to produce a live-birth than other women undergoing IVF and the chances of a twin birth increased with plasma and red cell folate concentrations [8]. The authors proposed that the woman's genotype is linked to her potential to produce good quality embryos and that this, coupled with folate status, increases the likelihood of IVF resulting in live births. Haggarty *et al *also observed an association between raised concentrations of homocysteine and an increase in the risk of miscarriage in their study of women undergoing IVF and elevated homocysteine levels has been linked recurrent pregnancy loss previously [10].

A meta-analysis has demonstrated that maternal hyperhomocysteinaemia (a metabolic consequence of low folate status) is associated with orofacial cleft and congenital heart defects [11], but the authors found no independent association between these congenital malformations and *MTHFR *genotype in either the mothers or children. However, the negative *in utero *effect of raised homocysteine has been proposed by Lucock and Yates as one of the mechanisms by which the genetic variant, *MTHFR *677C>T, compromises the viability of a pregnancy when dietary folate levels are inadequate [12]. These authors also hypothesise that when folate levels are unrestricted, there is a survival advantage *in utero *for homozygotes for the 677C>T allele because that genotype will increase the levels of 5,10 methylene tetrahydrofolate favouring DNA synthesis and stability. This hypothesis is supported by the authors of a recent study of the prevalence of this genotype in adult populations and in DNA samples taken from spontaneous abortions, they present evidence for the selection of the 677C>T allele associated with increased folate intake by women in the peri-conceptional period [13].

The *MTHFR *677C>T variant is associated with raised homocysteine concentrations and lower enzyme concentrations [14]. Association studies of the *MTHFR *677C>T variant and adverse pregnancy outcomes have in the main concluded that this variant is important in the pathogenesis of adverse pregnancy outcomes [15]. Less compelling evidence is available for *TYMS *genetic variants [16], one of which is a 5'UTR repeat variant (2R/3R) and the rare 2R variant is associated with lower TYMS expression and plasma homocysteine [17]. The simultaneous analysis of multiple genetic variants allows the consideration of additive, synergistic and compensating variants of folate metabolism. Disease association studies of two or more enzyme variants of folate metabolism are few, but suggest the possibility of gene-gene interactions or epistasis. One study has demonstrated a multiplicative effect on phenotype for the *MTHFR *677C>T and *MTRR *66G>A genotype combinations in relation to Down syndrome [4]. Another study demonstrated that three genotype combinations of the loci *MTRR/FOLH1*; *MTHFR *677/*CBS *and *MTHFR *677/*MTRR *increase the risk of neural tube defects [7].

A functional study of MTHFR and TYMS has shown that these enzymes compete for limiting supplies of folate required for homocysteine methylation [17]; which illustrates the likelihood that certain steps in one-carbon metabolism present bottlenecks for the distribution of folate species, and enzyme variants in these steps may be important.

We and others have previously found evidence for depletion of individuals homozygous for *MTHFR *677C>T relative to younger cohorts, and postulated it to be due to genotype-specific survival [18-20]. Because of observations made in cross-sectional population studies to explore the impact of genetic variants of the enzymes MTHFR and TYMS on survival in elderly cohorts, we have studied the prevalence of two polymorphisms in younger populations. The *MTHFR *and *TYMS *loci are found on chromosomes 1 and 18 respectively so linkage disequilibrium will not explain any digenic phenomena observed.

## Methods

### Subjects

The polymorphic locus *MTHFR *677C>T and the 5'UTR repeat variant of *TYMS *(2R/3R) were co-analysed in 2,898 DNA samples from the following 5 population cohorts.

#### Cohort 1

DNA extracted from 1178 blood samples collected from an elderly male population undergoing full blood counts in primary care or as patients at the Norfolk and Norwich University Hospital. Mean age = 84 years [range 70-99 years].

#### Cohort 2

DNA extracted from 438 blood samples collected from an elderly female population undergoing full blood counts in primary care or as patients at the Norfolk and Norwich University Hospital. Mean age = 92 years [range 89-104 years].

#### Cohort 3

DNA was extracted from 409 blood samples collected from a young female population undergoing full blood counts in primary care or as patients at the Norfolk and Norwich University Hospital. Mean age = 27 years [range 18-40 years].

#### Cohort 4

DNA was analysed from 398 blood samples from apparently healthy male volunteers recruited through a local community media-advertising campaign for a genetic and iron homeostasis study [21]. Mean age = 60 years [range 40-80 years]. The *MTHFR *data from this cohort has been described previously [22].

#### Cohort 5

DNA was obtained from 475 female volunteers from North Cumbria Community Genetics Project (NCCGP) [23]. Mean age of whole NCCGP cohort = 28 years [range 16-44 years].

Genotyping

#### DNA extraction and genotyping

High molecular weight DNA was sub-aliquoted onto 96 well plates at a concentration of approximately 100 ng/μl. All subsequent reactions were also performed in 96 well plates. The PCR reactions comprised of 100 ng DNA, 200 nmol/L of each primer and 1 × PCR Thermo Start Mastermix (ABgene UK, Epsom, England) in a 25 μl volume. The PCR conditions for each assay are shown in table 1.

**Table 1:** Description of PCR Assays

*GENE* SNP I.D. or description	*MTHFR* rs1801133	*TYMS* 5'UTR EX1+52CCGCGCCACTTC GCTGCCTCCGTCCCC
**Rare allelic form** **(amino acid changes)**	677C>T (A222V)	2R [17]

**Sense primer and antisense primer**	GGGTCAGAAGCATATCAGTCATG *CACAAAGCGGAAGAATGTGTC*	AAAAGGCGCGCGGAAG *GCCGGCCACAGGCAT*

**Annealing temperature, cycle number**	55°C, 38 cycles	61°C, 38 cycles

**PCR product size in base pairs**	326	111 or 139

**Restriction enzyme (number of units) or size of ins/del**	Hinf I (0.2), buffer 2	28 bp repeat visible by gel electrophoresis

**Fragment sizes for common allele in base pairs**. ***Fragment sizes for rare allele in base pairs***.	104 and 222 102, 166 and 53	139 111

The *MTHFR *assay required restriction digestion prior to gel electrophoresis. 10 μl of PCR product was digested overnight at 37°*Cina*20 μl reaction volume. The enzyme and buffer used (New England Biolabs, Hitchin, UK) are described in table 1.

The PCR products were electrophoresed on a 1 × Tris/Borate/EDTA, 3% Metaphor agarose (FMC Bioproducts, Lichfield, UK) gel in a stretch-wide apparatus (ABgene, Epsom, UK) at 80 V for 50 minutes.

#### Quality assurance

Gel analysis data were screened independently by two scientists (GW, BJ) and approximately 10% of the samples were genotyped a second time to check for concordance. A duplication of the *MTHFR *genotyping for 264 samples showed 100% concordance. A duplication of the *TYMS *genotyping for 362 samples showed 99.2% concordance and resulted in 3 reassigned genotypes. Furthermore, 4 samples that carried the rare 4 repeat allele were excluded from the analysis. The overall success rate for genotyping (in primary genotyping and re-analyses) was 99.6% for the *MTHFR *locus and 97.2% for the *TYMS *locus.

The samples from cohorts 1 to 4 were also genotyped for *MTHFR *1298 (rs1801131) and found to be in complete linkage disequilibrium with *MTHFR *677 as expected.

### Statistics

Hardy-Weinberg equilibrium (HWE); The χ^2^-test was used to analyse the frequencies of genotypes detected for each locus and relative fitness ratios were estimated for homozygotes compared to heterozygotes.

We tested the hypothesis that the proportion of individuals homozygous for the 2R allele of the 5'UTR *TYMS *polymorphism was lower in the group homozygous for the T allele of *MTHFR 677 *than in the group homozygous for the C allele of *MTHFR 677 *using a one-sided Fisher's exact test for each population. We used Stouffer's consensus combined P-value test, as described and recommended in Rice (1990), which is designed to combine the P-values from a set of independent tests addressing the same hypothesis [24].

### Ethics

Ethical approval for the study of anonymised DNA samples of groups 1 to 4 was obtained from Norwich and Waveney LREC. Ethical approval for the study of group 5 was obtained from Leeds West MREC.

## Results

### Genotyping

We have simultaneously determined the *MTHFR *677 and *TYMS *5'UTR genotype frequencies using DNA samples from 5 population cohorts comprising 2,898 individuals. The prevalence data and deviations from HWE are described in table 2 in addition to the relative fitness ratios for homozygotes compared to heterozygotes. Data are only presented for those samples that were successfully genotyped at both loci and which carried either the 2R or 3R allele at the *TYMS *locus.

**Table 2:** The *MTHFR *and *TYMS *genotype distributions, relative fitnesses and deviations from HWE of the genotypes in the 5 populations from Norfolk and Cumbria

*MTHFR*		*CC*	*CT*	*TT*	*Relative fitness* *CC vs CT*	*Relative fitness* *TT vs CT*	*Deviation from HWE*
Cohort 1	Observed	486.0	564.0	128.0	0.92	0.85	0.063
	Expected	500.7	534.6	142.7			
Cohort 2	Observed	206.0	191.0	41.0	0.98	0.95	0.824
	Expected	207.5	187.9	42.5			
Cohort 3	Observed	179.0	178.0	52.0	1.06	1.11	0.445
	Expected	175.6	184.8	48.6			
Cohort 4	Observed	166.0	202.0	30.0	0.81	0.61	**0.003**
	Expected	179.1	175.8	43.1			
Cohort 5	Observed	185.0	214.0	76.0	1.08	1.13	0.287
	Expected	179.5	225.0	70.5			

***TYMS***		**3_3**	**2_3**	**2_2**	**Relative fitness** **3_3 vs 3_2**	**Relative fitness** **2_2 vs 3_2**	**Deviation from HWE**

Cohort 1	Observed	350.0	541.0	287.0	1.16	1.18	**0.007**
	Expected	326.8	587.3	263.8			
Cohort 2	Observed	126.0	216.0	96.0	1.02	1.01	0.848
	Expected	125.0	218.0	95.0			
Cohort 3	Observed	129.0	180.0	100.0	1.24	1.28	**0.022**
	Expected	117.3	203.5	88.3			
Cohort 4	Observed	124.0	184.0	90.0	1.14	1.16	0.189
	Expected	117.2	197.6	83.2			
Cohort 5	Observed	146.0	240.0	89.0	0.96	0.94	0.641
	Expected	149.0	234.1	92.0			

Observed and expected genotype frequencies and deviations from HWE were calculated from the prevalence data for the *MTHFR *677 and *TYMS *5'UTR loci in 2898 samples that were simultaneously genotyped at both loci

**Figure 1:**
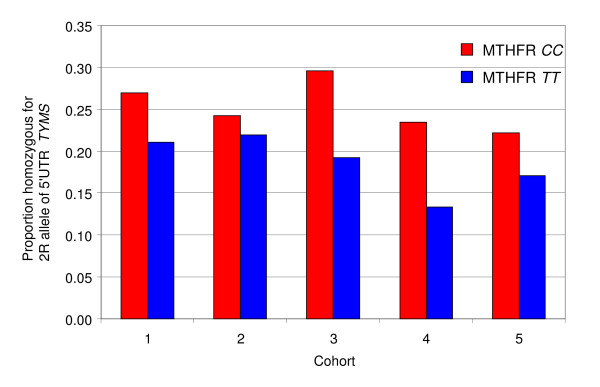
**Differences in distributions of the *TYMS *2R_2R homozygous genotype for each of the homozygous *MTHFR *genotypes**.

We observed that the proportion of individuals who are homozygous for the *TYMS *2R allele is different in the two groups of homozygotes for the *MTHFR *677; the proportion of individuals who are homozygous for the 2R allele of the 5'UTR *TYMS *polymorphism in individuals with the C>T allele of *MTHFR *677 is less than the proportion of individuals who are homozygous for the 2R allele in homozygotes for the C allele of *MTHFR *677 (see figure 1). We first made this observation in the elderly Norfolk cohorts 1 and 2. Therefore, to test if this observation was a real phenomenon, we analysed the *MTHFR *and *TYMS *genotypes in new populations and subsequently made the same observation in Norfolk cohorts 3 and 4 and in cohort 5, the female volunteers from North Cumbria Community Genetics Project [23]. The observations were consistent in all populations studied.

### Statistics

We found for all populations tested that the proportion of individuals who are homozygous for the *TYMS *2R allele was lower in the TT homozygotes for *MTHFR *677 than the CC homozygotes, but the individual tests were not significant (Table 3). The consensus combined p-value test [23] was significant (p = 0.02). Removing the hypothesis-generating cohorts 1 and 2 from the analysis, the consensus p-value was still significant (p = 0.04) for cohorts 3, 4 and 5 only.

**Table 3:** Distribution of *TYMS *homozygote status for *MTHFR *homozygotes

	***TYMS***									
					
	**Cohort 1**		**Cohort 2**		**Cohort 3**		**Cohort 4**		**Cohort 5**	
***MTHFR***	***2_2***	***Other***	***2_2***	***Other***	***2_2***	***Other***	***2_2***	***Other***	***2_2***	***Other***
					
*CC*	131	355	50	156	53	126	39	127	41	144
*TT*	27	101	9	32	10	42	4	26	13	63
					
P*		0.11		0.46		0.09		0.16		0.23
*Consensus P*^† ^0.02 (all cohorts), 0.04 (cohorts 3, 4 and 5 only)										

* One-sided Fisher's exact test

^† ^Obtained using Stouffer's consensus combined P-value test [24]

## Discussion

We have observed a consistent gene-gene interaction in 5 different cohorts. Certain genotypes are differentially represented in these populations and this could be evidence of genetic selection during early development; in gamete formation or *in utero*, with some gametes or pregnancies more or less viable because of variation in the way folate is handled at the cellular level, the metabolism of folate being the link between these enzymes and their genetic loci.

There is published evidence for significant genome-wide transmission distortion [25] which could be caused by genetic and/or environmental influences on gamete selection and embryo viability. Ebisch *et al *demonstrated that sperm concentration can be raised with folic acid and zinc sulphate supplementation, but only in males who are homozygous for the 677C genotype [26]. Lucock and Yates have hypothesised that when folate levels are unrestricted, there is a survival advantage *in utero *for homozygotes for the 677C>T allele because that genotype will increase the levels of 5,10 methylene tetrahydrofolate favouring DNA synthesis and stability [12]. The investigations by Mayor-Olea *et al *found that the wild type, 677CC, genotype is almost absent in spontaneous abortions suggesting that this genotype may protect against pregnancy loss. However, they also interpret the increase in the prevalence of the T allele alongside the use of peri-conceptional folic acid as an indication that an environmental factor can lead to the genetic selection for the T allele or permit the survival of deleterious genotypes [13].

Our observation could not be caused by any simple genotyping error because the relationship observed between *MTHFR *and *TYMS *is independent of heterogeneity in the allele frequencies for the individual polymorphisms between the cohorts. During the course of these experiments we investigated the possibility of technical artefacts because it can be seen from table 2 that some of the individual gene frequencies were out of HWE (see *MTHFR *frequency for cohort 4 and *TYMS *frequencies for cohorts 1 and 3) and genotyping errors could explain this. Undigested PCR product in the analysis of the *MTHFR *677 polymorphism, and preferential amplification of the *TYMS *allele with 2 rather than 3 repeats could produce erroneous data for the individual genotypes. Although only a single laboratory assay was used for each genotype analysis, the DNA quality and data interpretation checks described in the methods provide confidence in the reproducibility of our methods. Futhermore, while genotyping errors could result in perturbation of HWE they would not generate the significant co-segregation of *MTHFR *and *TYMS *genotypes described.

Deviation from HWE is also possible if there are selective advantages for particular genotypes. A selective advantage for particular *MTHFR *genotypes could lead to deviations from HWE in the general population; heterozygote advantage occurs when environmental situations lead to heterozygous individuals for some disease alleles having increased fitness over both homozygous genotypes. In the older cohorts (1, 2 and 4) in this study both homozygous groups showed a reduced fitness relative to the heterozygotes for *MTHFR *677 (see table 2). We and others have previously found evidence for depletion of individuals homozygous for the *MTHFR *677C>T relative to younger cohorts, and postulated it to be due to genotype-specific fatal disease [18-20,24]. However, given that the interaction between *MTHFR *and *TYMS *is found in all age groups, this interaction cannot be interpreted as an age-related survival factor; i.e. the death of some individuals from genotype-specific fatal disease does not explain the observation.

Given that all of the subjects from our study were born before the influence of folate related public health initiatives, could B-vitamin supplementation affect the biological phenomena that we and others have described? Haggarty *et al *report that folic acid intervention has not resulted in genetic selection in favour of the *MTHFR *677T allele [27], however this does not take into account potential gene-gene interactions at other folate-related loci and does not discount the possibility that numerous genetic variants may act in concert to exert selective pressure.

## Conclusions

Our data may suggest a gene-gene interaction and could be evidence of genetic selection, with some pregnancies more or less viable as a consequence of genetic variation. If these genetic phenomena affect the way folate is handled at the cellular level *in utero *it is possible that maternal folic acid intake may over-ride such genetic selection. Our findings should now be tested in independent population cohorts.

## Abbreviations

HUGO: approved nomenclature used for the gene loci; CBS: cystathionine-beta-synthase; *FOLH1*: folate hydrolase (prostate-specific membrane antigen) 1; *MTHFR*: 5,10-methylenetetrahydrofolate reductase (NADPH); *MTRR*: methyltetrahydrofolate-homocysteine methyltransferase reductase; *TYMS*: thymidylate synthetase; HWE: Hardy-Weinberg Equilibrium.

## Competing interests

The authors declare that they have no competing interests.

## Authors' contributions

GW, BJ and CR participated in the design of the study and contributed to the drafting or preparation of the manuscript. GW designed and optimised most of the PCR assays, collected and analysed most of the data. BJ also analysed the PCR data. CR also analysed the genotyping data by HWE. JS advised on and completed the statistical analyses. All authors contributed to and approved the final manuscript.

## Pre-publication history

The pre-publication history for this paper can be accessed here:

http://www.biomedcentral.com/1471-2350/11/18/prepub
